# Effects of Species and Structural Diversity on Carbon Storage in Subtropical Forests

**DOI:** 10.3390/biology15010079

**Published:** 2025-12-31

**Authors:** Liyang Tong, Yixuan Wang, Zhengxuan Zhu, Zhe Chen, Shigang Tang, Xueyi Zhao, Kai Chen, Lijin Wang

**Affiliations:** 1 College of Materials and Energy Engineering, Lishui University, Lishui 323000, China; 2 College of Agriculture and Biotechnology, Lishui University, Lishui 323000, China; 3 Zhejiang Lishui Ecological Environment Monitoring Center, Lishui 323000, China; 4 Zhejiang Environmental Monitoring Engineering Co., Ltd., Hangzhou 310012, China

**Keywords:** subtropical forest, biodiversity, carbon sequestration, forest type, species diversity, stand structure, carbon storage

## Abstract

Global CO_2_ concentrations are gradually increasing, and forests, as the main terrestrial carbon pool, are attracting growing attention in mitigating climate change. However, the impacts of forest types, species diversity, structural diversity, and environmental factors on the carbon sequestration mechanisms of subtropical forests remain unclear. This study established 45 forest plots (20 m × 20 m) in Lishui City, aiming to investigate the relationships between forest diversity, environmental factors, and carbon storage of subtropical forests among different forest types. Results showed that coniferous forests had the lowest species diversity (0.86), which exhibited extremely significant differences from broad-leaved forests (1.47, *p* < 0.01) and coniferous broad-leaved mixed forests (1.58, *p* < 0.01). The carbon storage of broad-leaved forests was 97.50 t·ha^−1^, which was higher than that of coniferous broad-leaved mixed forests (77.08 t·ha^−1^) and coniferous forests (75.57 t·ha^−1^). The carbon storage of coniferous forests was significantly positively affected by species diversity (*p* < 0.05). Tree height was the most significant structural diversity factor affecting forest carbon storage (*p* < 0.05). The results of the structural equation model (SEM) showed that the proportion of broad-leaved trees in forests and structural diversity had a significant positive effect on carbon storage (*p* < 0.01). Species diversity had a non-linear relationship with carbon storage. The ecological niche complementarity effect and selection effect interacted with changes in species diversity. When the species diversity was lower than 1.12 (Shannon–Wiener index), the ecological niche complementarity effect dominated and promoted carbon sequestration; when it was above this threshold, the selection effect dominated and weakened carbon sequestration. This study recommends prioritizing the planting of broad-leaved tree species during afforestation and paying attention to the current status of forest diversity.

## Simple Summary

Climate change remains a critical focus in ecological research, and forests play a key role in storing carbon and slowing this process. However, it is still unclear how different forest structures and combinations of tree species affect the amount of carbon stored in forests. To better understand this, we studied several forest areas in Lishui City. We measured forest diversity, species types, and species numbers and calculated the carbon stock. We found that the greater the proportion of broad-leaved trees in a forest, the higher its carbon storage capacity. Our study showed that both niche complementarity and selection effects influenced carbon sequestration, with these two effects exhibiting an interactive relationship. When species diversity was low, niche complementarity enhanced forest carbon sequestration; when species diversity was high, selection effects diminished forest carbon sequestration. This study recommends prioritizing the planting of broad-leaved tree species during afforestation and paying attention to the current status of forest diversity.

## 1. Introduction

Human activities have led to elevated levels of carbon dioxide and other greenhouse gases in the atmosphere, which are contributing to climate change [1]. As the primary terrestrial reservoir for CO_2_, forests play an indispensable role in mitigating climate change [2,3,4]. As climate change intensifies, forest carbon storage has attracted widespread attention and become a research focus [1,5,6,7]. Currently, forests cover more than 30% of the Earth’s land surface [8,9]. Forest ecosystems serve not only as major terrestrial carbon sinks but also as the largest carbon reservoirs [6,7,9,10]. Studies have shown that increasing forest cover and biomass can effectively enhance carbon storage and reduce atmospheric carbon concentrations, making this one of the most significant strategies for alleviating global climate change [11,12]. Therefore, exploring strategies to increase forest carbon stocks has emerged as a central challenge in addressing the potential impacts of climate change [11].

Recent studies have demonstrated that forest types exhibit distinct patterns of carbon storage capacity across various geographical regions [13]. In temperate zones, coniferous forests typically store more tree biomass carbon than broad-leaved forests [14]. Conversely, in tropical regions, broad-leaved forests generally possess significantly higher carbon stocks than coniferous forests [15]. These differences may be attributed to species-specific adaptive traits, as coniferous species are primarily distributed in mid- to high-latitude regions, whereas broad-leaved species are more prevalent in low- to mid-latitude regions. While current research has predominantly focused on temperate and tropical forests, subtropical ecosystems have remained comparatively understudied. As a critical component of the global carbon cycle, subtropical forests play a vital role in mitigating global climate change [13]. Furthermore, there is considerable heterogeneity in the findings of subtropical forest studies. For instance, Wu et al. [13] found that broad-leaved trees in subtropical forests demonstrated higher carbon storage efficiency than coniferous trees, whereas Li et al. [16] suggested that coniferous species may outperform broad-leaved species in terms of carbon storage capacity. Existing studies have not clearly identified the non-linear relationship between species diversity and carbon storage in subtropical forests and the threshold effect, nor have they adequately explained the mechanism of the effect of stand structure diversity on the ecosystem. Therefore, there is a pressing need for further research on the carbon dynamics of subtropical forest ecosystems.

In addition to the type of forest, forest carbon storage is strongly influenced by the community structure and the species diversity [17,18,19], with structurally complex forests typically having greater carbon storage capacity [20]. The influence of biodiversity on forest carbon storage is primarily determined by two ecological mechanisms: niche complementarity and the selection effect [13]. The niche complementarity effect posits that differences in species’ ecological niches allow more complete occupation of available resource space, thereby enhancing resource use efficiency and forest productivity [21]. The selection effect, on the other hand, refers to the dominance of highly productive or functionally important species that positively influence ecosystem functioning [13,22]. While previous studies have confirmed that biodiversity plays a critical role in sustaining ecosystem functions in grassland systems, the mechanisms through which biodiversity affects forest ecosystems remain more complex [11,12,23]. These two mechanisms may act independently or interactively across different forest types or structural compositions to influence carbon storage [21]. Thus, further investigation is imperative to ascertain the relationship between forest biodiversity and carbon sequestration.

This study focuses on subtropical forests, aiming to enhance our understanding of the relationship between forest diversity and carbon storage in these ecosystems. Specifically, the study focuses on how the Shannon–Wiener index (H) affects the carbon stocks of coniferous forests, mixed forests, and broad-leaved forests and explores the actual impact of broad-leaved trees on forest carbon stocks and the underlying ecological mechanisms involved. In subtropical forests, we hypothesized that (1) broad-leaved tree species are more favorable for carbon accumulation than conifers; (2) forest diversity or structural diversity produce different influencing mechanisms on carbon stocks with different forest types; and (3) interactions exist between niche complementarity and selection effects in forests.

## 2. Materials and Methods

### 2.1. Study Area and Field Investigation

The study site was located in Lishui City, situated in the southwestern part of Zhejiang Province, China (27°25′–28°95′ N, 118°41′–120°26′ E). The terrain is dominated by hills and low mountains, with a total area of approximately 1.73 × 10^4^ km^2^. The region experiences a typical mid-subtropical monsoon climate, characterized by warm and humid conditions, abundant rainfall, and distinct mountain climate features [24]. The forest types at the study site included coniferous forest (CF), coniferous and broad-leaved mixed forest (MF), and broad-leaved forest (BF). A total of 45 plots were chosen, with 15 plots for each forest type (Table 1).

The forest plots measured were 20 × 20 m [13]. All woody plants with a diameter at breast height (DBH) ≥ 5 cm within each subplot were recorded for DBH, tree height (TH), and crown length (CW). Field surveys were conducted from August to October 2024. Species identification was carried out based on the Catalogue of Life China: 2023 Annual Checklist [25].

### 2.2. Diversity Indices and Environmental Variables

We utilized the H to represent plant species diversity based on the relative abundance of species [26]. Stand structural diversity was represented by the coefficients of variation (CVs) of DBH (CV_DBH_, %), tree height (CV_TH_, %), and crown width (CV_CW_, %) for each plot [13,27]. The Shannon–Wiener index was calculated by the following formula:

(1) $$H=-\sum \left(P\mathrm{i}\right)\left(\ln P\mathrm{i}\right)$$

where *Pi* indicates the proportion of individuals of a given species relative to the total number of individuals in the community.

This study utilized five environmental factors, including altitude, slope, aspect, mean annual temperature (MAT), and mean annual precipitation (MAP). Altitude, slope, and aspect were measured using RTK and a compass in forest plots, and MAT and MAP were sourced from the WorldClim database (http://www.worldclim.org). We used ArcGIS Pro 3.2.0 (Environmental Systems Research Institute, Inc., Berkeley, CA, USA, Reference ID: 1156174312) to obtain the MAT and MAP data for each forest plot. We used principal component analysis (PCA) to analyze environmental factors [13].

### 2.3. Carbon Stock Estimation

Aboveground biomass was estimated using allometric growth equations (Table S1). For species lacking specific equations, a general genus- or family-level equation was applied, and no previously unrecorded species were identified during the survey. Carbon stocks were calculated from biomass, and species-specific carbon fractions are provided in Table S2 [24].

### 2.4. Statistical Analysis

Linear regression models were employed, and a least significant difference (LSD) test (*p* < 0.05) was used to assess statistical differences among forest types. A structural equation model (SEM) was employed to investigate the effects of environmental factors and forest diversity on carbon storage. We employed linear regression models to analyze the correlations among structural diversity factors and forest carbon stocks, and only those forest structural diversity factors that were significantly correlated with carbon stocks were included in the SEM modeling. We used the “piecewiseSEM” package to fit the SEM; the fitting criteria for the SEM were *p* > 0.05, a comparative fit index (CFI) > 0.95, a standardized root mean square residual (SRMR) < 0.08, and a root mean square error of approximation (RMSEA) < 0.05 [13]. Before regression analyses, the normality of the data was assessed, and normality was improved using the “bestnormsize” package (Table S3). All variables were standardized using Z-scores to allow for comparisons across parameter estimates. All analyses were conducted using R-4.5.3.

## 3. Results

### 3.1. Environmental Factors, Forest Biological Diversity, and Carbon Storage

**Figure 1:**
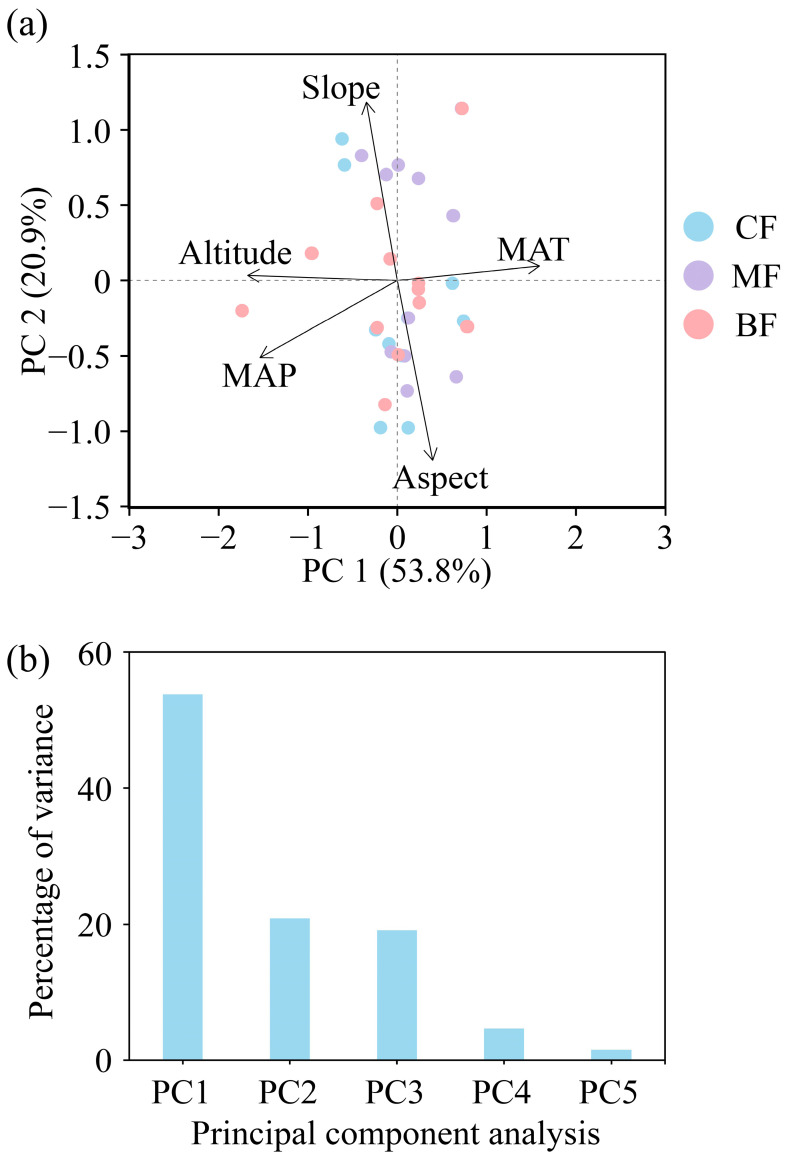
Principal component analysis (PCA) plot of environmental factors. (**a**) Environmental factors trait, (**b**) PCA percentage of variance. CF, coniferous forest; MF, coniferous and broad-leaved mixed forest; BF, broad-leaved forest; MAT, mean annual temperature; MAP, mean annual precipitation.

This study used PCA to analyze environmental factors (Figure 1a) and used the first axis (environmental PC1, Env PC1) and the second axis (environmental PC2, Env PC2) of PCA as predictors for subsequent analyses (Table 2). The results showed that the total explanatory power of the two axes reached 74.70% (Figure 1b), among which the PC1 axis represented slope gradient and aspect, while the PC2 axis represented altitude, MAT, and MAP.

**Figure 2:**
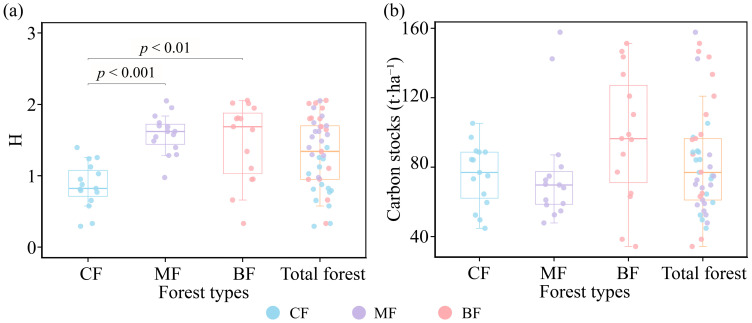
Species diversity and carbon storage in different forest types. (**a**) Species diversity (calculated with the H index) in different forest types, (**b**) carbon storage in different forest types. CF, coniferous forest; MF, coniferous and broad-leaved mixed forest; BF, broad-leaved forest. Significant results were marked in the figure; non-significant results were not marked.

This investigation showed that the Shannon–Wiener index ranged from 0.29 to 2.06 in the study area. The Shannon–Wiener index differed significantly among the three forest types (Figure 2a). CF differed significantly from both BF and MF (*p* < 0.01), with the Shannon–Wiener indices of BF (1.47) and MF (1.58) both being higher than that of CF (0.86).

The results showed that mean carbon storage was 83.38 t·ha^−1^ in forest plots (Figure 2b). The average carbon storage for BF (97.50 t·ha^−1^) was higher than that of MF (77.08 t·ha^−1^) and CF (75.57 t·ha^−1^). The coefficient of variation in carbon storage for CF (0.24) was lower than that for MF (0.41) and BF (0.38), and the CF data showed minimal variation (Table 3).

### 3.2. Effects of Forest Diversity on Carbon Storage

**Figure 3:**
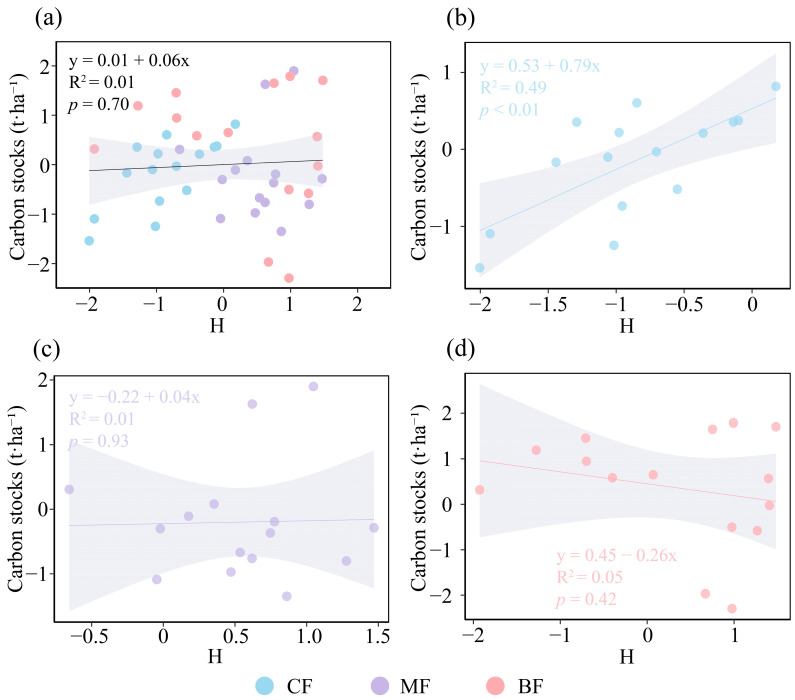
Relationships between species diversity (calculated with the Shannon–Wiener index) of community and carbon stocks under different forest types. (**a**) Coniferous forest, (**b**) coniferous and broad-leaved mixed forest, (**c**) broad-leaved forest, (**d**) total forest. The solid lines represent the fitted regression line, accompanied by the coefficient of determination (*R*^2^) and *p* value. The gray shading indicates the 95% confidence band. CF, coniferous forest; MF, coniferous and broad-leaved mixed forest; BF, broad-leaved forest. All variables were *Z*-score transformed prior to analysis.

**Figure 4:**
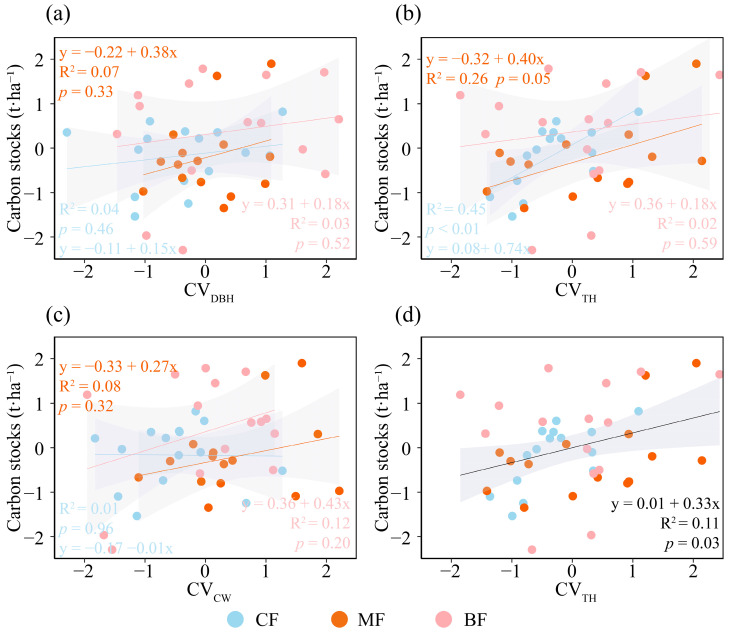
Relationships between structural diversity (calculated with CV_DBH_, CV_TH_, and CV_CW_) of community and carbon stocks under different forest types. (**a**) Relationship between CV_DBH_ and carbon storage in different forest types, (**b**) relationship between CV_TH_ and carbon storage in different forest types, (**c**) relationship between CV_CW_ and carbon storage in different forest types, (**d**) relationship between CV_TH_ and carbon storage in total forest. The solid lines represent the fitted regression line, accompanied by the coefficient of determination (*R*^2^) and *p* value. The gray shading indicates the 95% confidence band. CF, coniferous forest; MF, coniferous and broad-leaved mixed forest; BF, broad-leaved forest. All variables were *Z*-score transformed prior to analysis.

The results concerning the relationship between species diversity and forest carbon storage indicated that, in the overall forest analysis, species diversity did not exert a significant influence on forest carbon storage (Figure 3a). In CF, species diversity showed a significantly positive association with forest carbon storage (*p* <0.01, Figure 3b); in MF, species diversity did not have a significant effect on forest carbon storage (Figure 3c); and in BF, species diversity exhibited a negative association with forest carbon storage (Figure 3d). The relationship between structural diversity and carbon stocks varied among forest types. CV_DBH_ demonstrated a modest positive influence on carbon storage across all three forest types (Figure 4a); CV_TH_ exerted a significant positive effect on carbon storage in CF and MF, while showing a moderate positive influence in BF (*p* ≤ 0.05, Figure 4b); and CV_CW_ had a modest positive impact on carbon storage in all forest types (Figure 4c). Overall, CV_DBH_ and CV_CW_ exhibited modest but non-significant positive effects on forest carbon storage, whereas CV_TH_ showed a comparatively stronger and statistically significant positive effect (*p* ≤ 0.05, Figure 4d). Therefore, CV_TH_ was selected as the structural diversity indicator for the SEM.

### 3.3. Driving Factor Analysis of Forest Carbon Storage

**Figure 5:**
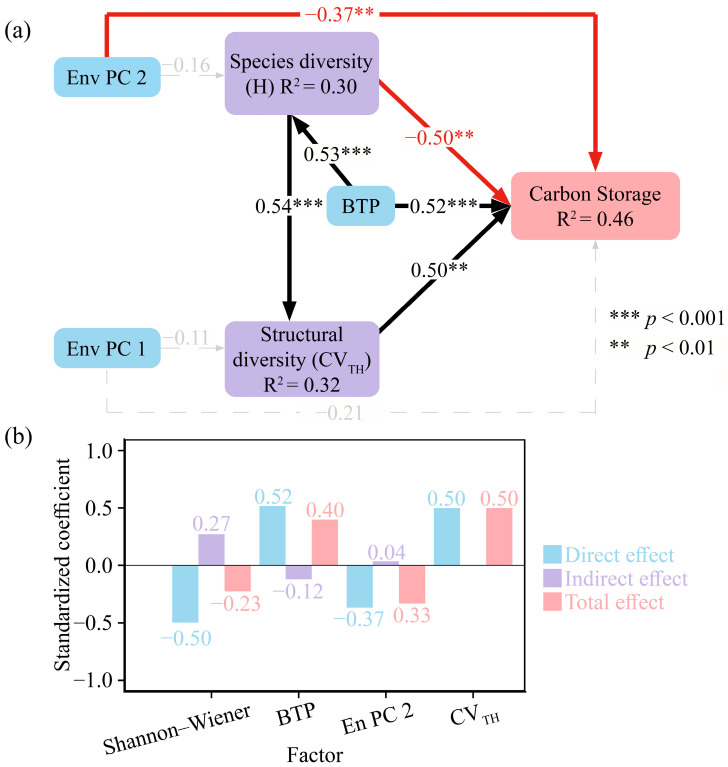
Structural equation model presenting the effects of environmental variables, broad-leaved tree proportion, plant species diversity, and stand structural diversity on carbon stocks. (**a**) Path diagrams of factors influencing changes in carbon stocks, (**b**) total direct and indirect effects combined. Env PC1, environmental PC1; Env PC2, environmental PC2; H, Shannon–Wiener index; BTP, broad-leaved tree proportion; CV_TH_, coefficient of variation in tree height. All variables were *Z*-score transformed prior to analysis.

The SEM had a good fit (Table 4), explaining 46% of the variance in changes to carbon stocks (Figure 5a). The results of the structural equation model showed that both broad-leaf tree proportion, plant species diversity, and stand structural diversity influenced forest carbon stocks. The broad-leaf tree proportion and stand structural diversity (CV_TH_) had a significantly positive effect (*p* < 0.01) on carbon storage, with path coefficients of 0.52 and 0.50, respectively, while the species diversity had a significantly negative effect, with a path coefficient of −0.50. Furthermore, Env PC2 (slope and aspect) directly influenced (*p* < 0.01) the forest carbon stocks, with a path coefficient of −0.37.

This study further analyzed the indirect effects and total impact effects of different variables on carbon stocks (Figure 5b). The results showed that plant species diversity had a positive indirect effect of 0.27 on carbon stocks. The indirect effect stems from the fact that species diversity, by influencing the proportion and structural diversity of broad-leaved trees, exerts an indirect effect on carbon storage. Combined with the direct effect and the indirect effect, it resulted in a total effect of −0.23 of plant species diversity on carbon stocks.

**Figure 6:**
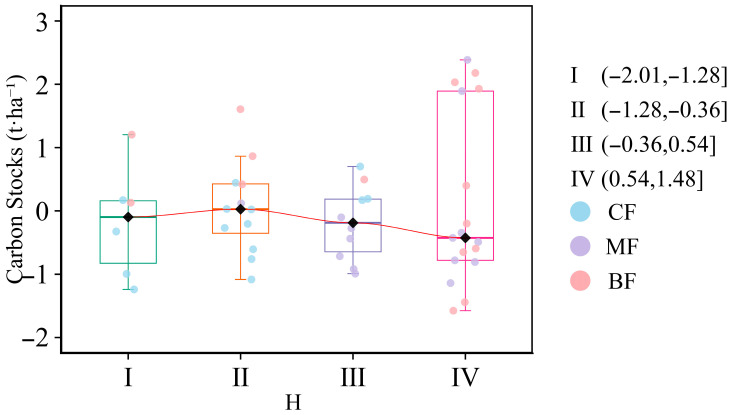
Impact of the Shannon–Wiener index from the natural breaks method (Jenks) on carbon stocks. I, II, III, and IV were the natural breaks method (Jenks) classification for the Shannon–Wiener index. CF, coniferous forest; MF, coniferous and broad-leaved mixed forest; BF, broad-leaved forest. All variables were *Z*-score transformed prior to analysis.

To analyze the negative correlation between species diversity and carbon stocks, the Shannon–Wiener index was segmented into four types (I, II, III, and IV) using the natural breaks method (Jenks), and the impacts of different Shannon–Wiener index values on carbon stocks were studied separately (Figure 6, Table S4). The results showed that when the Shannon–Wiener index was less than 1.12, carbon stocks exhibited an overall upward trend. After exceeding 1.12, carbon stocks showed an overall downward trend. Only 40% of forest plots exhibited an upward trend, while 60% exhibited a downward trend.

## 4. Discussion

### 4.1. Broad-Leaved Trees and Forest Carbon Storage

We calculated the carbon stocks of three forest types, and the results supported our first hypothesis: broad-leaved forests exhibited higher carbon stocks than the other two forest types (Figure 2a). Other studies have reported similar findings in subtropical forests [13,28]. The results of the structural equation model (SEM) indicated that a higher proportion of broad-leaved trees was associated with greater forest carbon storage (Figure 5a). This outcome may be attributed to broad-leaved trees having a larger leaf area index, higher photosynthetic efficiency, and faster decomposition rate of litter, promoting nutrient cycling and biomass accumulation, thereby increasing carbon storage [29]. In addition, species with diverse functional traits may utilize ecosystem resources more efficiently [30]. It is therefore recommended that broad-leaved species be prioritized in forest management to effectively increase forest carbon stocks. Additionally, our research found that broad-leaved forests and coniferous and broad-leaved mixed forests exhibit relatively high CV values for carbon stocks, which may be related to their species composition [30,31], as broad-leaved trees include both hardwoods and softwoods. Although these trees are all classified as broad-leaved trees, their carbon sequestration capacity varies, which may influence forest carbon stocks [20]. This factor was not considered in the present study. The data might have had certain limitations, but this approach aligned better with natural conditions. Future research should place greater emphasis on distinguishing among different broad-leaved tree types.

### 4.2. Mechanism of Impact on Forest Carbon Storage

The findings of this study demonstrated that forest structure influences carbon storage, with higher forest structural diversity correlating with greater forest carbon stocks. Among the structural factors examined, CV_TH_ emerged as the most significant variable, exerting a pronounced effect on forest carbon storage. A high coefficient of variation in tree height indicated a more complex vertical structure of the forest stand, which could better utilize resources such as light and water, indicated the total photosynthetic capacity of the community, and reflected the complementary effect of ecological niches [32]. We attributed this primarily to enhanced complementarity in trees’ utilization of vertical structural resources, which boosted forest photosynthesis [17]. Photosynthesis plays a significant role in the accumulation of biomass [33] and hence can significantly affect ecosystem carbon sinks; this represented the coordinated utilization of resources and embodied the principle of ecological niche complementarity [21,34]. Recent research has found that CV_TH_ exhibited a significant effect on carbon storage, whereas CV_DBH_ and CV_CW_ had no significant impact [13]. The reason for this might be that the impacts of tree diameter at breast height and crown width on carbon storage are characterized by a delay [35]. Higher CV_DBH_ and CV_CW_ have relatively minor effects on forest coverage, as they require longer timeframes and larger scales to manifest their effects [36], while a larger CV_TH_ may create gaps in the forest structure, facilitating sunlight penetration into the forest, and the increased light intensity within the forest further promotes carbon storage [37]. Due to scale limitations, only slope gradient and aspect showed significant effects on forest carbon stocks. This might indicate that at the scale of this study, ecosystems exhibited reduced sensitivity to variations in other environmental factors.

Current research suggests that actively modifying species diversity may yield immediate changes in forest carbon sinks [32]. There is ample support for positive biodiversity–ecosystem function relationships in forest studies performed [13,28,31]. The influence of biodiversity on forest carbon storage was primarily determined by two ecological mechanisms: niche complementarity and the selection effect [30,38]. However, our study showed that with the increase in biodiversity, there was a certain transition process between these two mechanisms. We argue that resources in each community are limited; before resources are fully utilized, the increase in diversity can enable more efficient resource utilization to enhance forest productivity, and niche complementarity dominates in this scenario [34]. And once this saturation point is exceeded, species with similar characteristics and functions would be redundant in the existing environment, reducing the positive impact of species diversity on community productivity, and the selection effect dominates in this case [39]; species competition might even reverse productivity [33]. Consistent with our third hypothesis, the results of the structural equation model (SEM) also supported this view, demonstrating that biodiversity exerted both negative and positive effects on carbon storage, and this negative correlation was the result of a threshold effect. At low diversity, species diversity still promoted carbon sinks; at high diversity, due to species functional redundancy and intensified competition, carbon sinks decreased. After the Shannon–Wiener index was classified using the Jenks method, the diversity saturation point for the communities in this study was found to be 1.12. However, our study showed that the proportion of broad-leaved trees, species diversity, structural diversity, and environmental factors have a relatively low overall impact on forest carbon storage (*R^2^* = 0.46). We believe this may be attributed to the failure to consider complete root system data and soil data. Although the root-to-stem ratio (R:S) of subtropical forests is significantly lower than that of other forests (0.23 ± 0.02) [40], another reason is the lack of integration of soil data, and current studies have shown that soil nitrogen content affects carbon storage [13]. Due to its small scale and lack of integration with soil carbon data and complete root system data, this research still had limitations. Future studies should focus on expanding the research scale and considering factors such as tree species, root system, and soil conditions to enhance the accuracy of experimental results.

## 5. Conclusions

This study examined the mechanisms driving carbon storage in subtropical forests. The findings demonstrate that forest carbon stocks are jointly determined by tree species composition, stand structural diversity, and species diversity. Specifically, a higher proportion of broad-leaved trees increased carbon storage. Stand structural diversity, particularly the variation in tree height, exhibited a positive relationship with carbon accumulation. Moreover, the relationship between species diversity and carbon storage was non-linear, characterized by a threshold value (Shannon–Wiener index) of 1.12. Below this threshold, niche complementarity dominated, enhancing carbon sequestration; above it, the selection effect became dominant, weakening carbon sequestration.

These results highlight that attention should be paid to the current status of forest biodiversity, and broad-leaved trees should be prioritized for planting during afforestation to enable forests to achieve higher carbon sequestration efficiency. Future research should prioritize expanding the research scale and incorporating more environmental factors to enhance the accuracy of experimental results.

## Figures and Tables

**Table 1:** Detailed information of the study sites.

Study Site	CF	MF	BF
Number of sample plots	15	15	15
Longitude (°)	118.80–120.10	118.84–120.12	119.03–120.15
Latitude (°)	27.73–28.64	27.50–28.71	27.50–28.57
Forest age (years)	28 ± 3	26 ± 4	30 ± 4
Stand density (trees/ha)	1918	1801	1911
Mean annual temperature (°C)	15.90	15.99	15.53
Mean annual precipitation (mm)	1691	1658	1673

Abbreviations: CF, coniferous forest; MF, coniferous and broad-leaved mixed forest; BF, broad-leaved forest.

**Table 2:** Component loadings and eigenvalues of principal components (PCs) chosen from PCA on environmental factors.

Environmental Factors	EnvPC1	EnvPC2
Altitude (m)	−0.59	0.02
Mean annual temperature (°C)	0.56	0.05
Mean annual precipitation (mm)	−0.54	−0.29
Slope (°)	−0.12	0.67
Aspect (°)	0.14	−0.68
Cumulative proportion explained	0.54	0.75

**Table 3:** Carbon stocks and Shannon–Wiener index (average and coefficient of variation) of different forest types.

Forest Types	Carbon Stocks		Shannon–Wiener	
	Average	CV	Average	CV
Coniferous forest	75.57	0.24	0.86	0.37
Coniferous and broad-leaved mixed forest	77.08	0.41	1.58	0.17
Broad-leaved forest	97.50	0.38	1.47	0.37
Total forest	83.38	0.37	1.31	0.39

Abbreviations: CV, coefficient of variation.

**Table 4:** Model fit statistic summary of the tested SEM for carbon stocks.

Model Fit Statistics Summary	Fit Statistic
CFI	1.00
SRMR	0.05
RMSEA	0.01
AIC	342.96
χ^2^ (*p*-value)	0.39
*R* ^2^	0.46

Abbreviations: CFI, comparative fit index; SRMR, standardized root mean square residual; RMSEA, root mean square error of approximation; AIC, Akaike’s information criterion; χ^2^, chi-square test; *R^2^* indicates the total variation in carbon stocks.

## Data Availability

The datasets generated and/or analyzed during the current study are available in the Supplemental Text. The climate data (mean annual temperature and mean annual precipitation) in this study were accessed through the WorldClim database (http://www.worldclim.org/) (accessed on 6 April 2023).

## Supplementary Materials

The following supporting information can be downloaded at https://www.mdpi.com/article/10.3390/biology15010079/s1, Table S1: Biomass model table. Table S2: Carbon contents of species. Table S3: Data normality test results. Table S4: The natural breaks method (Jenks) classification table.

## Abbreviations

CF	Coniferous forest	CV_CW_	Coefficient of variation in crown width
MF	Coniferous and broad-leaved mixed forest	PCA	Principal component analysis
BF	Broad-leaved forest	PC	Principal component
H	Shannon–Wiener index	LSD	Least significant difference
MAT	Mean annual temperature	SEM	Structural equation model
MAP	Mean annual precipitation	CFI	Comparative fit index
DBH	Diameter at breast height	SRMR	Standardized root mean square residual
TH	Tree height	RMSEA	Root mean square error of approximation
CW	Crown length	Env PC1	Environmental PC1
CV	Coefficient of variation	Env PC2	Environmental PC2
CV_DBH_	Coefficient of variation in diameter at breast height	R^2^	Coefficient of determination
CV_TH_	Coefficient of variation in tree height		
