# Supplementary material for: Effects of Species and Structural Diversity on Carbon Storage in Subtropical Forests
Source: Biology (Basel). 2025 Dec 31;15(1):79. doi: 10.3390/biology15010079 (PMC12784675; doi:10.3390/biology15010079)
Supplement: Supplementary file 1 [file biology-15-00079-s001.zip › biology-4023869-supplementary.pdf]

Effects of Species and Structural Diversity on Carbon Storage in Subtropical Forests

Liyang Tong [1,2], Yixuan Wang [3], Zhengxuan Zhu [4], Zhe Chen [2], Shigang Tang [1], Xueyi Zhao [1], Kai Chen [1,2] and Lijin Wang [1,*]

[1] *College of Materials and Energy Engineering, Lishui University, Lishui 323000, China*

[2] *College of Agriculture and Biotechnology, Lishui University, Lishui 323000, China*

[3] *Zhejiang Lishui Ecological Environment Monitoring Center, Lishui, Zhejiang 323000, China*

[4] *Zhejiang Environmental Monitoring Engineering Co.Ltd, Hangzhou, Zhejiang 310000, China*

* Correspondence: lsxywlj@126.com (Lijin. Wang)

Supplementary material

Table

Table S1

Biomass model table.

Tree Species Type	Tree Species Classification	Biomass Model	Reference Source
Coniferous trees	Pinaceae	$B_{Pinaceae} = B_1 + B_2 + B_3$	[24]
		$B_1 = 0.0600\, H^{0.7934}\, D_{BH}^{1.8005}$	
		$B_2 = 0.1377\, L^{0.4052}\, D_{BH}^{1.4873}$	
		$B_3 = 0.0417\, H^{-0.0780}\, D_{BH}^{2.2618}$	
	Cupressaceae	$B_{Cupressaceae} = B_1 + B_2 + B_3$	
		$B_1 = 0.0647\, H^{0.8959}\, D_{BH}^{1.4880}$	
		$B_2 = 0.0971\, L^{0.0346}\, D_{BH}^{1.7814}$	
		$B_3 = 0.0617\, H^{-0.1037}\, D_{BH}^{2.1153}$	
Broadleaf trees	Hardwood I	$B_{Hardwood\ 1} = B_1 + B_2 + B_3$	
		$B_1 = 0.0560\, H^{0.8099}\, D_{BH}^{1.8140}$	
		$B_2 = 0.0980\, L^{0.4610}\, D_{BH}^{1.6481}$	
		$B_3 = 0.0549\, H^{0.1068}\, D_{BH}^{2.0953}$	
	Hardwood II	$B_{Hardwood\ 2} = B_1 + B_2 + B_3$	
		$B_1 = 0.0803\, H^{0.7815}\, D_{BH}^{1.8056}$	
		$B_2 = 0.2860\, L^{0.9450}\, D_{BH}^{1.0968}$	
		$B_3 = 0.2470\, H^{0.1745}\, D_{BH}^{1.7954}$	

Softwood	$B_{Softwood} = B_1 + B_2 + B_3$<br>$B_1 = 0.0444\, H^{0.7197}\, D_{BH}^{1.7095}$<br>$B_2 = 0.0856\, L^{0.3970}\, D_{BH}^{1.2266}$<br>$B_3 = 0.0459\, H^{0.1067}\, D_{BH}^{2.0247}$
Bamboo	$B_{Bamboo} = B_1 + B_2 + B_3$<br>$B_1 = 0.0398\, H^{0.5778}\, D_{BH}^{1.8540}$<br>$B_2 = 0.2800\, L^{0.2740}\, D_{BH}^{0.8357}$<br>$B_3 = 0.3710\, H^{0.1357}\, D_{BH}^{0.9817}$

Abbreviations: H represents tree height (m), D_{BH} denotes diameter at breast height (cm), and L indicates crown length (m). B_1, B_2, and B_3 correspond to the stem biomass (kg), crown biomass (kg), and root biomass (kg) of an individual tree (or bamboo), respectively. For biomass calculation, individual trees were classified into six species types: Pinaceae, Cupressaceae, Hardwood I, Hardwood II, Softwood, and Bamboo.

Table S2

Carbon contents of species.

Species	Carbon contents	Reference Source
Cunninghamia lanceolata	0.52	[24]
Pinus massoniana	0.46	
Cryptomeria japonica	0.52	
Cinnamomum camphora	0.49	
Sassafras tzumu	0.48	
Quercus	0.50	
Other	0.50	[18]

Table S3

Data normality test results.

Factor	Shapiro-Wilk p			
	CF	MF	BF	Total
Carbon stocks	0.26	0.03	0.09	0.75
Shannon-Wiener	0.86	0.95	0.06	0.07
CV_{DBH}	0.80	0.62	0.23	0.36
CV_{H}	0.83	0.40	0.54	0.27
CV_{P}	0.81	0.48	0.05	0.86

Table S4

The natural breaks method (Jenks) classification table.

Grade	Shannon-Wiener	Number of Plots
I	(-2.01, -1.28]	6
II	(-1.28, -0.36]	12
III	(-0.36, 0.536]	10
IV	(0.536, 1.48]	17

References

18. Fatimata, N.; Philippe, M.; Bienvenu, S.; Nicole, F. Exploring the effects of forest management on tree diversity, community composition, population structure and carbon stocks in sudanian domain of Senegal, West Africa. *For. Ecol. Manag.* **2024**, *559*, 121821. https://doi.org/10.1016/j.foreco.2024.121821.

24. Wang, J.W.; Ji, B.Y.; Wang, Z.Y.; Zhu, C.H. Impact of subtropical forest landscape pattern on forest carbon density in Lishui City of Zhejiang Province. J. Zhejiang AF Univ. 2024, 41, 30–40. https://doi.org/10.11833/j.issn.2095-0756.20230205.
